# 2010 Use it but still lose it: Exploring age-related changes in skeletal stem cell location and activation in response to physical stimulation

**DOI:** 10.1017/cts.2018.146

**Published:** 2018-11-21

**Authors:** Pamela C. Zuckerman, Chao Liu, Alesha B. Castillo

**Affiliations:** 1 H+H Clinical and Translational Science Institute, New York University, New York, NY, USA; 2 New York University, New York, NY, USA; 3 Veterans Affairs New York Harbor Healthcare System, New York, NY, USA

## Abstract

OBJECTIVES/SPECIFIC AIMS: Our goal is to assess age-related changes in osteogenic stem cell populations of bone tissue. We hypothesize that aging mice have reduced osteogenic capacity in response to physical stimulation due to aging-associated decline in osteoprogenitor cell number and their proliferative capacity. METHODS/STUDY POPULATION: Mechanical loading: The NYU School of Medicine Institutional Animal Care and Use Committee approved all procedures. The response of tibial periosteal cells to physical stimulation or mechanical loading was assessed in 16-week-old adult (n=6) and aged 78-week-old female (n=4) mice subjected to 4 consecutive days of strain-matched axial compressive loading (1400 μm, 120 cycles, 2 Hz). Whole Mount Staining: Baseline periosteal cell numbers and nuclear morphology were assessed by whole bone DAPI staining of the antero-medial region of the tibiae in adult and aged mice (n=6). Immunohistochemistry: Tibiae were fixed in 4% PFA, decalcified in 19% EDTA, OCT-embedded, and thickly sectioned (150 μm) at midshaft. Sca1+, Prrx1+, and Ki67+cell numbers were quantified by simultaneous fluorescent immunohistochemical staining from loaded and nonloaded contralateral tibiae. Nonimmune species specific serum served as negative controls. Imaging: 3D image datasets of the periosteum at the antero-medial region of the tibial midshaft were acquired by multi-photon and confocal microscopy. Quantification of Sca1+, Prrx1+, and Ki67+ cells was carried out using Particle Analysis software (ImageJ) and Imaris 7.4.2 Surface Rendering Statistics functions. Cell number was normalized to periosteal area (~0.04 mm^2^). A Student *t*-test determined significance at *p*<0.05. RESULTS/ANTICIPATED RESULTS: At baseline, aged periosteal cell nuclei (DAPI+) area (14% decrease, *p*<0.0001), nuclei number, and Prrx1+ cell number (22% decrease) was significantly lower compared with adult mice. In loaded adult mice, Prrx1+but not Sca1+cell number increased significantly (35%, *p*=0.0115). Proliferating Sca1+(top panel) and Prrx1+(top panel) cells also increased with loading, 62%, *p*=0.0253 and 115%, *p*=0.0004, respectively, in adult but not aged mice. The percentage of Prrx1+ cells undergoing proliferation (co-expressing Ki67+) in the total Prrx1+ cell population increased significantly with loading (bottom panel). Aged mice did not exhibit significant differences in loaded versus nonloaded controls for all other outcomes. Our data suggest fundamental changes in periosteal cell morphology, number and response to mechanical loading with aging. The significant increase in total Prrx1+ cell number and the number of Prrx1+ cells undergoing proliferation with loading in adult mice, suggest that the Prrx1+ cell population expands through proliferation. In fact, loading resulted in a 2-fold increase in the percentage of Prrx1+ preosteogenic cells undergoing proliferation. Accordingly, the significant age-related decrease in Prrx1+ cells may explain, in part, the attenuation of load-induced bone formation in aged mice. Loading resulted in greater numbers of proliferating Sca1+ cells (the more primitive cell) in adult mice, though this represented only a small percentage (<10%) of the total Sca1+ population. Mechanical loading expands the Prrx1+ pre-osteogenic cell population, but not the more primitive Sca1+ population. However, this load-induced osteogenic effect in the periosteum is not observed in aged mice, which may explain age-related diminishment of load-induced bone formation. DISCUSSION/SIGNIFICANCE OF IMPACT: Mechanical loading presents an inexpensive treatment for increasing bone mass and bone strength, but may be insufficient to prevent or reverse age-related bone loss due to reduced numbers of osteogenic progenitors in the periosteum. Therapeutic approaches targeting the osteogenic capacity of periosteal cells will be required to address declining mechanoresponsiveness with age.

